# Atrial Fibrillation-Induced Cardiomyopathy in Scimitar Syndrome With Electrically Silent Anomalous Pulmonary Vein: An Adult Case Report

**DOI:** 10.1016/j.cjcpc.2024.11.002

**Published:** 2024-11-19

**Authors:** Takahiko Kinjo, Fumitake Miura, Maiko Senoo, Shingo Sasaki, Yuichi Toyama, Shota Washima, Hirofumi Tomita

**Affiliations:** a Department of Cardiology and Nephrology, Hirosaki University Graduate School of Medicine, Hirosaki, Japan; b Department of Pediatrics, Hirosaki University Graduate School of Medicine, Hirosaki, Japan; c Department of Advanced Management of Cardiac Arrhythmias, Hirosaki University Graduate School of Medicine, Hirosaki, Japan; d Department of Cardiac Remote Management System, Hirosaki University Graduate School of Medicine, Hirosaki, Japan; e Department of Advanced Therapeutics for Cardiovascular Diseases, Hirosaki University Graduate School of Medicine, Hirosaki, Japan

**We describe the case of a 52-year-old man with isolated scimitar syndrome and atrial fibrillation (AF)-induced cardiomyopathy. Left ventricular ejection fraction was normalized 8 months after persistent AF was abolished through catheter ablation. The pulmonary blood flow (Qp) to systemic blood flow (Qs) ratio (Qp/Qs) was 1.37 upon the initial assessment; after treatment, Qp/Qs decreased to 1.11 because of improved Qs, whereas Qp remained unchanged. This report highlights the importance of rhythm control in patients with congenital heart disease. Treating heart failure and arrhythmia before deciding on the indications for surgery is essential.**

Scimitar syndrome is a rare variation of partial anomalous pulmonary vein (PV) connection (PAPVC),^1^ in which the right PV connects to the inferior vena cava (IVC). Although scimitar syndrome is often associated with right lung hypoplasia and other congenital cardiac anomalies, cases of isolated scimitar syndrome have also been reported.^2^ In general, a pulmonary blood flow (Qp)/systemic blood flow (Qs) ratio of >1.5 and evidence of right ventricular (RV) volume overload are indications for surgical repair of PAPVC.^3^

Here, we describe a case of isolated scimitar syndrome with arrhythmia-induced cardiomyopathy due to atrial fibrillation (AF). Our case highlighted a potential pitfall wherein arrhythmia-induced cardiomyopathy due to AF can overestimate Qp/Qs even in patients with extracardiac left-to-right shunts.

## Case Description

**Figure 1:**
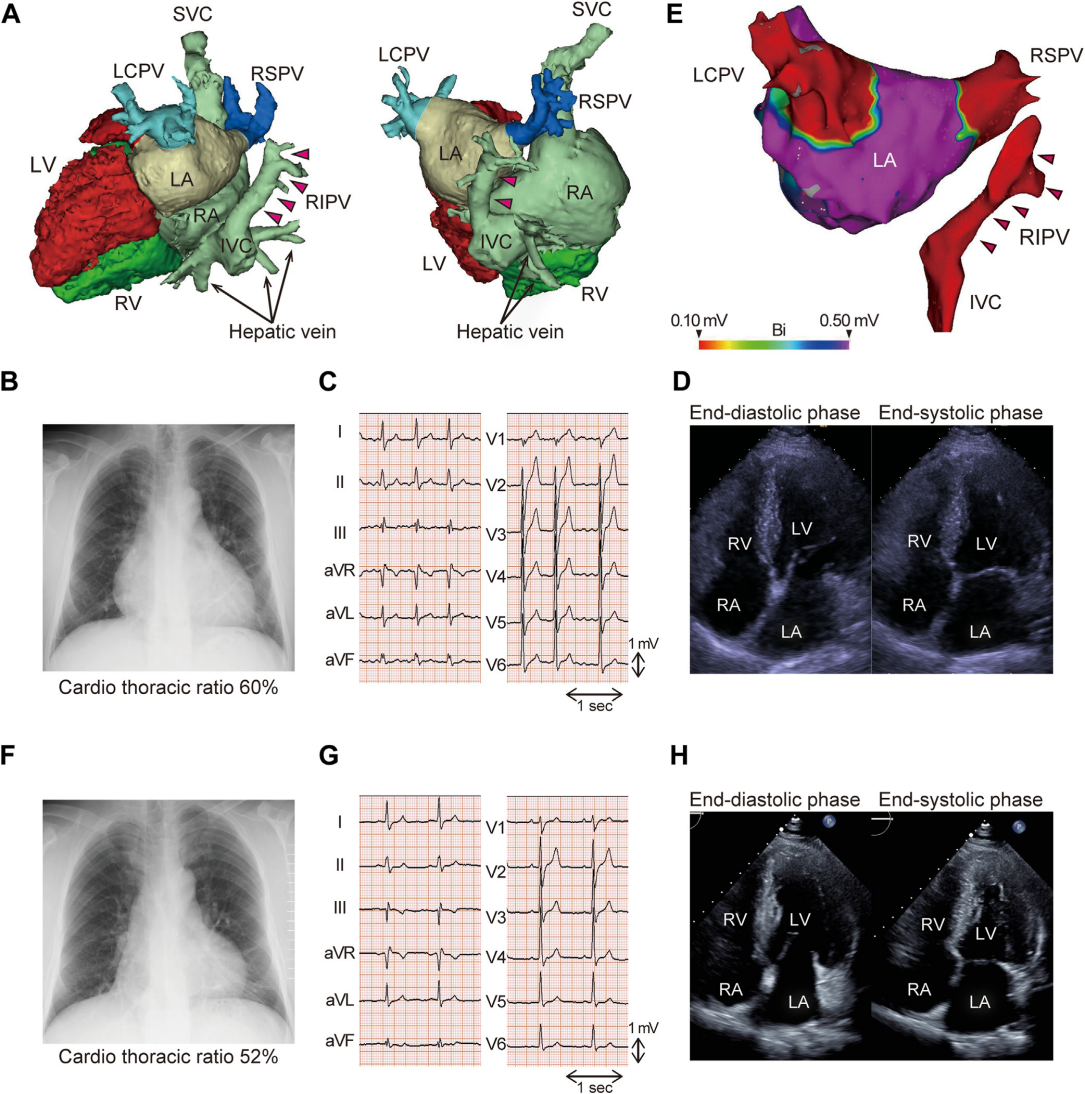
(**A**) Reconstructed 3-dimensional computed tomography. Left panel: modified left lateral view. Right panel: modified posterior view. The right inferior pulmonary vein (RIPV) connects to the inferior vena cava (**arrows coloured in magenta**). (**B-D**) The findings upon referral to our hospital. (**B**) Chest radiograph showing enlargement of the cardiac shadow. (**C**) Electrocardiography showing atrial fibrillation with a heart rate of 101 beats/min. (**D**) The LV ejection fraction (LVEF) is 34.7%, the left ventricular end-diastolic (LVDd) is 56.6 mm, and the basal right ventricular end-diastolic diameter (RVDd) is 35.9 mm. (**E**) Posterior-to-anterior view of a bipolar voltage map after pulmonary vein isolation under atrial pacing from the distal coronary sinus. LA had no low-voltage zone (0.1-0.5 mV). The RIPV showed no electrical activity (**arrows coloured in magenta**). (**F**-**H**) The findings 7 months after pulmonary vein isolation. (**F**) Chest radiograph showing improvement in enlarged cardiac shadow. (**G**) Electrocardiography showing sinus rhythm with a heart rate of 66 beats/min. (**H**) LVEF is 59.0%, LVDd is 47.0 mm, and basal RVDd is 27.1 mm. IVC, inferior vena cava; LA, left atrium; LCPV, left common pulmonary vein; LV, left ventricle; RA, right atrium; RSPV, right superior pulmonary vein; RV, right ventricle; SVC, superior vena cava.

A 52-year-old Japanese man was referred to our department for persistent AF ablation. He was hospitalized for decompensated heart failure and persistent AF 8 months before referral. He had no significant medical history before this episode and had not undergone regular medical checkups; therefore, the onset and duration of AF was undetected. He was diagnosed with heart failure with reduced left ventricular ejection fraction (LVEF) and persistent AF. Coronary artery disease was ruled out using coronary angiography. After the acute-phase treatment, the patient was treated with bisoprolol (5 mg once daily), valsartan (80 mg once daily), eplerenone (25 mg once daily), furosemide (40 mg once daily), rivaroxaban (15 mg once daily), and amiodarone (100 mg twice daily). However, no improvement in the LVEF was observed. The patient returned to sinus rhythm once direct-current cardioversion was performed, but the AF recurred. Contrast-enhanced computed tomography before AF ablation revealed PAPVC in which the right inferior PV (RIPV) was connected to the IVC (Fig. 1A). He was then diagnosed with isolated scimitar syndrome.

At the time of referral, chest radiography revealed an enlarged heart (Fig. 1B), electrocardiography showed AF (Fig. 1C), and echocardiography showed left ventricular dilation and low LVEF (Fig. 1D), whereas RV dilation was not significant. The rest heart rate was 101 beats/min even though the rate control was tried using bisoprolol (5 mg once daily) and amiodarone (100 mg twice daily). Blood tests revealed elevated N-terminal pro–B-type natriuretic peptide (Table 1). Echocardiography suggested RV dysfunction with a fractional area change of 25% and a tricuspid annular plane systolic excursion of 13 mm. Transthoracic and transesophageal echocardiography and contrast-enhanced computed tomography ruled out any other congenital cardiovascular anomalies or hypoplasia of the lungs. An endomyocardial biopsy revealed no specific findings, suggesting secondary cardiomyopathy.

**Table 1:** Cardiac catheterization and blood sampling data

	At initial examination	Seven months after rhythm control
Pressure (mm Hg)		
RA (mean)	15	6
RV (systolic/edp)	39/14	32/7
PA (systolic/diastolic/mean)	37/21/29	30/12/19
PAWP (mean)	17	6
LV (systolic/edp)	–	123/7
Ascending aorta (systolic/diastolic/mean)	–	136/75/100
O_2_ saturation (%)		
SVC	62.8	70.5
IVC∗	70.7	85.9
RA	68.9	71.5
RV	70.3	–
Main PA	71.3	73.1
Ascending aorta†	94.2	96.5
Heart rate (beats/min)	98	55
Qp (L/min/m^2^)	2.74	2.69
Qs (L/min/m^2^)	2.00	2.42
Qp/Qs	1.37	1.11
PVRI (wood unit m^2^)	4.38	4.83
SVRI (wood unit m^2^)	–	38.8
PVRI/SVRI	–	0.124
Haemoglobin (g/dL)	16.5	15.8
NT-proBNP (pg/mL)	2021	97

edp, end-diastolic pressure; IVC, inferior vena cava; LV, left ventricle; NT-proBNP, N-terminal prohormone of brain natriuretic peptide; PA, pulmonary artery; PAWP, pulmonary arterial wedge pressure; PVRI, pulmonary vascular resistance index; Qp, pulmonary blood flow; Qs, systemic blood flow; RA, right atrium; RV, right ventricle; SVC, superior vena cava; SVRI, systemic vascular resistance index.

∗ Due to an oxygen saturation step-up in the IVC, the mixed venous oxygen saturation was substituted with the oxygen saturation in the SVC.

† The oxygen saturation of the blood obtained from the aorta was used as a surrogate for pulmonary vein saturation.

Circumferential PV isolation (PVI) was performed to abolish or dissociate the electrical activity of the left common PV and right superior PV via radiofrequency catheter ablation using the CARTO3 system (Biosense Webster; Johnson & Johnson, Irvine, CA) under unsedated state. The RIPV connected to the IVC showed no electrical activity (Fig. 1E). After PVI, sinus rhythm was restored via direct-current cardioversion. No AF recurrence due to non-PV triggers was observed. The procedure time and left atrial dwell time was 95 minutes and 59 minutes, respectively. Radiofrequency energy was applied 49 times, and saline needed for catheter irrigation was 340 mL. Subsequent cardiac catheterization revealed a Qp/Qs ratio of 1.37 (Table 1). The patient was diagnosed with pulmonary hypertension associated with left heart disease because the pulmonary arterial wedge pressure was >15 mm Hg.

Seven months later, cardiac size decreased (Fig. 1F), sinus rhythm maintained without amiodarone (Fig. 1G), LVEF normalized (Fig. 1H), and N-terminal pro–B-type natriuretic peptide levels decreased (Table 1). RV dysfunction was also improved; fractional area change was 33%, and tricuspid annular plane systolic excursion was 19 mm. Furosemide was discontinued, and cardiac catheterization results showed resolution of pulmonary hypertension and decreased Qp/Qs (Table 1); thus, surgery was not performed. Two years after PVI, there was no worsening of heart failure nor recurrence of AF.

## Discussion

This report highlights 2 important clinical implications. The first is the importance of AF ablation in patients with adult congenital heart disease (ACHD), because arrhythmia-induced cardiomyopathy due to AF can occur. The second is the potential pitfall in which arrhythmia-induced cardiomyopathy can overestimate Qp/Qs, even in patients with extracardiac left-to-right shunts.

This report suggests the importance of treating heart failure, including performing AF ablation in patients with ACHD. The patient was diagnosed with arrhythmia-induced cardiomyopathy due to AF because the LVEF normalized after eliminating AF.^4^ The treatment of arrhythmias as part of heart failure therapy is emphasized in the current guideline for ACHD.^3^ The importance of AF ablation in patients with ACHD is expected to increase because the number of patients is growing, and the incidence of AF increases with age. In this case, we isolated only the PVs connected to the left atrium, owing to the lack of electrical activity in the RIPV connection to the IVC. Because myocardial sleeves of the IVC can be a source of AF,^5^ it may be necessary to verifying whether there is potential in the PV connected to the IVC. Reports on AF ablation in patients with PAPVC^6^ are limited. Previous case series described that electrical activity of anomalous PV was observed in 5 of 9 patients, and isolation of anomalous PV was performed in 2 patients; meanwhile, it is unclear whether an AF-triggering premature atrial complex was seen from the anomalous PV.^6^ Although it is of great interest whether electrically active anomalous PV can cause AF, further investigation is warranted.

This report emphasizes the potential pitfall in which overestimation of Qp/Qs is caused by LV dysfunction even in patients with only extracardiac left-to-right shunts. In the present case, after LVEF recovered, the Qp/Qs decreased from the initial 1.37 to 1.11. This was due to improved Qs, while Qp remained unchanged and/or reduced left atrial/ventricular pressure. The magnitude of shunting through an anomalous PV connection vs normally draining PVs is determined by respective outflow resistances.^7^ In patients with PAPVC with left-sided heart failure, as the left atrial/ventricular pressure increases > right atrium pressure, the Qp/Qs ratio increases as blood follows the path of least resistance to the right atrium through the anomalous PV. The opposite phenomenon would have occurred after the treatment of heart failure and reduced left atrial pressure. In this case, the Qp/Qs ratio was not indicative of surgical intervention at both the initial and follow-up examinations; however, in some instances, Qp/Qs might cross 1.5 before and after treatment, posing a potential pitfall in determining the indication for surgery. It is crucial to carefully decide on the indications for surgery because most adult patients with scimitar syndrome have a good prognosis without surgical correction.^3^

There are several limitations in this report. First, catheter ablation may influence the subsequent cardiac catheterization data, although the ablation was performed with the patient unsedated and fasting for 6 hours, and the saline infusion was only 340 mL. Second, the cardiac magnetic resonance was not performed because the use of gadolinium-based contrast agents was hesitated based on the presence of chronic kidney disease (estimated glomerular filtration rate was 47 mL/min/1.73 m^2^). We would have considered cardiac magnetic resonance if the patient had no improvement in LV function after rhythm control. Third, the pulmonary vascular resistance index (PVRI) was high even after normalized LVEF (Table 1). The systemic vascular resistance index (SVRI) at the follow-up catheterization was 38.3 wood unit m^2^; therefore, the PVRI/SVRI ratio was 0.124, which is not considered high. The SVRI was high during the catheterization. Therefore, we did not consider high PVRI alone as a contraindication for surgical repair because pulmonary hypertension was not detected and PVRI is less than one-third of SVRI.

In conclusion, we describe a case of isolated scimitar syndrome in which arrhythmia-induced cardiomyopathy due to AF overestimated the Qp/Qs ratio. It is essential to treat heart failure, including rhythm control for AF, before deciding on the indications for surgical repair in patients with ACHD.

**Novel Teaching Points**

• We described an adult case of isolated scimitar syndrome in which arrhythmia-induced cardiomyopathy due to atrial fibrillation (AF) overestimated the pulmonary blood flow/systemic blood flow ratio.
• It is essential to treat heart failure, including efforts to achieve rhythm control for AF, before deciding on the indications for surgical repair in patients with adult congenital heart disease (ACHD).
• The patient maintained sinus rhythm after circumferential pulmonary vein (PV) isolation of the left common PV and right superior PV, both of which were connected to the left atrium; the right inferior PV connected to the inferior vena cava showed no electrical activity.
• The role of AF ablation is expected to increase in patients with ACHD because the number of patients is growing, and the incidence of AF increases with age. However, the indication, detailed strategy, efficacy, and safety of AF ablation for patients with ACHD remain to be elucidated.
